# Achieving safe and high-performance gastrointestinal tract spectral CT imaging with small-molecule lanthanide complex

**DOI:** 10.1186/s40824-023-00463-x

**Published:** 2023-11-22

**Authors:** Xiaoling Che, Chunmei Yang, Liping Pan, Didi Gu, Guidong Dai, Jian Shu, Lu Yang

**Affiliations:** Department of Radiology, The Affiliated Hospital of Southwest Medical University, Luzhou, 646000 People’s Republic of China

**Keywords:** Spectral CT, Lanthanide complex, CT contrast agent, Colitis, Gastrointestinal tract, CT imaging

## Abstract

**Background:**

Non-intrusive imaging of gastrointestinal (GI) tract using computed tomography (CT) contrast agents is of the most significant issues in the diagnosis and treatment of GI diseases. Moreover, spectral CT, which can generate monochromatic images to display the X-ray attenuation characteristics of contrast agents, provides a better imaging sensitivity for diagnose inflammatory bowel disease (IBD) than convention CT imaging.

**Methods:**

Herein, a convenient and one-pot synthesis method is provided for the fabrication of small-molecule lanthanide complex Holmium-tetraazacyclododecane-1, 4, 7, 10-tetraacetic acid (Ho-DOTA) as a biosafe and high-performance spectral CT contrast agent for GI imaging with IBD. In vivo CT imaging was administered with both healthy mice and colitis mice induced by dextran sodium sulfate.

**Results:**

We found that Ho-DOTA accumulated in inflammation sites of large intestines and produced high CT contrast compared with healthy mice. Both in vitro and in vivo experimental results also showed that Ho-DOTA provided much more diagnostic sensitivity and accuracy due to the excellent X-ray attenuation characteristics of Ho-DOTA compared with clinical iodinate agent. Furthermore, the proposed contrast media could be timely excreted from the body via the urinary and digestive system, keeping away from the potential side effects due to long-term retention in vivo.

**Conclusion:**

Accordingly, Ho-DOTA with excellent biocompatibility can be useful as a potential high-performance spectral CT contrast agent for further clinical imaging of gastrointestinal tract and diagnosis of intestinal system diseases.

**Graphical Abstract:**

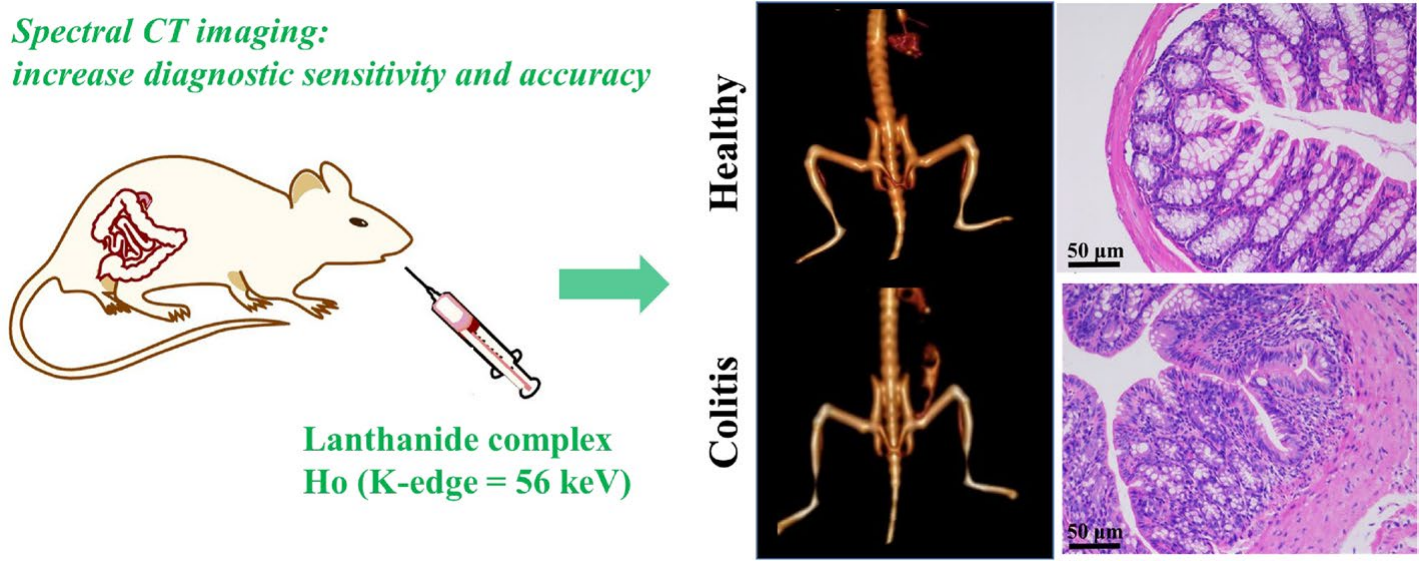

**Supplementary Information:**

The online version contains supplementary material available at 10.1186/s40824-023-00463-x.

## Introduction

Gastrointestinal diseases, as a highly diverse group of conditions, are clinically very common and affect an increasing number of humans, such as Cohn’s disease (CD) and ulcerative colitis [1, 2]. Visual inspection of gastrointestinal pathology and anatomy has become an important tool for the diagnosis and prognosis of GI diseases. At present, multiple imaging modalities are applied to diagnose these diseases in clinical and basic research, such as endoscopic techniques [3], fluorescence imaging [4, 5], X-ray imaging [6], computed tomography (CT) [7, 8], magnetic resonance imaging (MRI) [9, 10], single-photon emission computed tomography (SPECT) [11], photo acoustic imaging [12, 13], and multimodal imaging [14–17]. However, the endoscopic technique is invasive, which is suboptimal and unfavourable for patient follow-up study [18–21]. Moreover, due to the overlapping ultrasound signs of different intestinal diseases, routine ultrasound examination lacks the diagnostic accuracy to inflammatory bowel disease (IBD) [22]. MRI provides excellent spatial resolution while high cost and time-consuming [23]. X-ray and CT imaging have become the most widely used imaging modes to clinical diagnose of GI diseases for the advantages of simple imaging process, low price and no tissue damage in various non-intrusive imaging techniques [3, 24]. In particular, the rising energy spectrum CT technique, which can image an object using two X-ray sources with different properties, has attracted increasing attention [25, 26]. Energy spectrum CT can distinguish various kinds of tissues according to their different attenuation characteristics, generate mono-energy X-ray images representing single X-rays, reduce image artefacts, decrease the dose of contrast agent and radiation, opening up a novel and promising mean for gastrointestinal tract imaging [27, 28].

Currently, artificial contrast agents (CAs) and natural contrast agents are used in GI imaging [29]. The former, which can be present in the form of fruit juice, pulps or tea, have been found to have minimal side effects [30]. One limitation of these contrast agents is not widely available due to their growth feature [31, 32]. Artificial contrast agents have the advantages of wide availability, easy preparation process, low cost, and more general use for GI imaging [29]. Currently, there are two major categories in artificial contrast agents: small molecular contrast agents (iodinated molecules and lanthanide-chelates) [33–35] and nanostructures composed by high-Z elements [28, 36–38]. Due to obvious accumulation in the kidney and liver, these nanoparticles agents have some defects, including intrinsic extended retention, difficulty to biochemical degradation in vivo [39]. Thus, barium sulphate suspension and iodinated small molecules are commonly used clinically for GI imaging [29], but all do not have a characteristic diagnosis of inflammatory bowel disease. The barium sulphate is the contrast agent of choice for gastrointestinal angiography, but oral barium sulphate may have a risk of intestinal perforation, fecal impaction and constipation in patients with colitis [30]. In addition, the barium and iodine have a weak X-ray absorption capacity caused by a small atomic number and K edge value, so that these are not suitable for spectral CT imaging [25, 31]. Consequently, the discovery of novel spectral CT contrast agents for imaging the gastrointestinal tract and diagnosing the intestinal system diseases is urgent.

To overcome intrinsic limitations of clinical CAs, lanthanide complexes have been used to manufacture CAs for enhanced CT imaging due to outstanding X-ray absorption capacity [40–48], which greatly facilitates the development of spectral CT contrast agents. The lanthanide metal Ho has a K edge value of 56 keV [47], indicating that the Ho element has large X-ray attenuation capability (e. g., Ho: 3.49 cm ^2^ g ^−1^ and I: 1.94 cm ^2^ g ^−1^, at 100 keV), resulting in strong CT imaging capabilities. Although literatures have previously demonstrated the superiority of nanoparticle contrast agents based on Ho element in MRI [49, 50]. To our knowledge, the study of Ho-based small-molecule complex as spectral CT contrast agents for gastrointestinal tract imaging had not been reported. Thus, to take full advantage of the advanced sensitivity of spectral CT and K-edge energy of Ho (56 keV), small-molecule Ho complex was investigated as a novel spectral CT contrast agent to increase the imaging sensitivity of in vivo imaging in this study.

At present, chelating ligands have been widely used in biological research due to their easily preparation and modified characteristics, such as diethylenetriaminepentaacetic acid (DTPA) [33, 34] and 1,4,7,10-tetrazacyclicdodecane-1,4,4,7,10-tetraacetic acid (DOTA) [35]. However, DOTA with a ring-like structure has better chemical stability than DTPA and is easier to apply in clinical practice [51, 52]. Herein, the purpose of this study is to combine Ho element with chelate ligand DOTA to prepare lanthanide complex Ho-DOTA as a high-performance spectral CT contrast agent for GI imaging with or without IBD. Ho-DOTA was prepared by a facile and green one-pot method and its X-ray attenuation ability was verified by in vitro and in vivo experiments. Specially, the spectral CT imaging performance of Ho-DOTA in the diagnosis of colitis was studied further by constructing mouse models of colitis.

### Experimental section

#### Synthesis of Ho-DOTA complex

The Ho-DOTA was prepared using a minor modified literature protocol [33, 35]. Under argon atmosphere, Ho_2_O_3_ (1 mmol) and DOTA (2 mmol) were mixed in 50 mL DI water and reacted under vigorous stirring at 100 °C until forming a transparent solution. The obtained Ho-DOTA solution was treated with N-Methyl-D-glutamine to adjust the pH value to 7.2, and the product of Ho-DOTA was freeze dried for further study.

#### The structure stability of Ho-DOTA complex

To evaluate the colloidal stability of Ho-DOTA, which was dispersed in various medium including phosphate buffer saline (PBS), saline solution, fetal bovine serum (FBS), Roswell Park Memorial Institute-1640 medium (RPMI-1640) and Dulbecco’s modified eagle medium (DMEM) for 14 days at 37 °C, and their photos were taken at 7 and 14 days to monitor if there were precipitates or aggregates formed. To evaluate the chemical structure stability, the leakage of Ho^3+^ in Ho-DOTA solution was investigated by using the Xylenol Orange (XO) indicator (0.2%).

### Cell cytotoxicity assessment

The cell cytotoxicity in vitro was measured by CCK8 assay. We seeded MCF-10A cells and COS-1 cells into a 96-well cell culture plate at 10^6^/well and then incubated them for 24 h in a 5% CO_2_ and 37 ℃ environment. DMEM solution of Ho-DOTA with different concentrations (25, 50, 100, 200, 400, 600 μg/mL) were added to the wells for 24 h. After washing with PBS, we added 100 μL fresh DMEM solution and 10 μL of the CCK8 reagent into the wells. The absorbance at 450 nm of each well was measured using a micro plate reader for the cell viability calculations after a 2 h-incubation.

### Toxicity assessment in vivo

In order to assess the in vivo poisonousness of Ho-DOTA, the healthy mice were sacrificed at different time points (1 and 14 days, respectively) post injection (*n* = 6) and oral (*n* = 6). As a control, the other mice were administrated with normal saline (NS) and iohexol, and sacrificed at 1 and 14 days respectively. The important organs (heart, spleen, liver, lung, kidney post injection; stomach, intestine, and colon post oral) were discreetly harvested and fixed with 4% paraformaldehyde universal-type tissue fixative solution. Lastly, pathological section and haematoxylin and eosin (H&E) staining analysis were carried out to investigate the in vivo toxicity of Ho-DOTA. In addition, the body weight changes of the mice were measured every 2 days. Some important biochemical indicators (the liver and kidney function indicators) of these mice were determined at different time points (1 and 14 days, *n* = 3 for each group) after injecting Ho-DOTA (0.2 mol/L) or iohexol (0.2 mol/L). The liver function indicators mainly included Alanine aminotransferase (ALT) and Aspartate Transaminase (AST). The kidney function indicators mainly included UREA, Creatinine (CREA) and Uric Acid (UA).

### Spectral CT imaging in vitro

To investigate the CT imaging ability, we respectively prepared different concentrations of Ho-DOTA and iohexol solutions (0, 0.0125, 0.025, 0.05, 0.1 and 0.2 M Ho/I). Furthermore, all CT scanning were carried out on spectral CT (IQON Spectral CT, Philips, Netherlands) in our experiments. The parameter settings as follows: field of view 150 × 150 mm, slices thickness 0.4 mm, tube current 100 mA and tube voltage 120 kV. Thereafter, virtual monochromatic images of these solutions were obtained at the photon energy range of 40–150 keV with a 10-keV increment. The 3D reconstruction was performed on a Philips Intellispace Portal Workstation.

### The construction of acute dextran sodium sulphate colitis model

According to a published protocol [53], acute dextran sodium sulphate colitis (DSS-colitis) mice were modeled using dextran sodium sulphate (DSS). On day 1, at first, all mice were weighed and numbered, and then randomly divided into two groups. The water supply of the mouse cages was added into an appropriate amount of 5% (w / v) DSS (molecular weight: 36,000–50000 Da, MP Biomedical, Irvine, CA) to induce colitis. The control group received the purified water without DSS. Then, in experimental group, the remaining DSS solution was replaced with fresh DSS solution every 2 days. On day 8, the remaining DSS solution was replaced by purified water. After 8 days of DSS treatment, the mice developed colitis in their large bowel. Compared with healthy mice, colitis mice showed significant weight loss (Fig. S1).

### Spectral CT imaging in vivo

The 8-week-old BABL/C female mice (average body weight: 16 g) were purchased from Beijing HFK Bio-Technology. Co., Ltd (Beijing, China) and then were placed in an environment including 50% ± 5% humidity, 20–25 °C, a 12 h light–dark cycle and a freedom access to food and water. All procedures and animal experiments were approved by the Animal Ethnical Committee of Southwest Medical University (project number: 20221227–001) and conducted according to the Guide for the Care and Use of Laboratory Animals (8th edn) published by NIH. After normal feeding a week, all mice were an anesthetized by using a small animal ventilator with isoflurane (1.5–2.5%, 0.8 mL/min oxygen flow rate). Then healthy mice scanned before and after the injection of 200 μL Ho-DOTA (0.1 and 0.2 M, respectively) via the tail vein at different time points (0, 1 min, 5 min, 30 min, 1 h, 1.5 h, 3 h, 6 h and 12 h). The colitis mice and healthy mice were taken in the volumes (200 μL via oral) of 0.2 M Ho-DOTA solutions and scanned before and after oral Ho-DOTA solutions at different time points (Pre, 5 min, 30 min, 1 h, 2 h, 4 h, 12 h and 24 h).The spectral CT imaging of the GI tract performed with a tube voltage of 120 keV. As control, the healthy mice and colitis mice were treated with an equivalent dose of Ho-DOTA and iohexol, and their spectral CT images were collected under the same conditions. After 24 h of oral administration, the large intestine tissue from colitis and healthy mice were harvested and analyzed by ICP-OES after washing with PBS to determine the concentration of Ho element. Moreover, electron microscopy on large intestine tissue samples from DSS-induced colitis mice were acquired at 24 h post administration of Ho-DOTA.

## Results

### Synthesis and characterization of Ho-DOTA complex

**Fig. 1:**
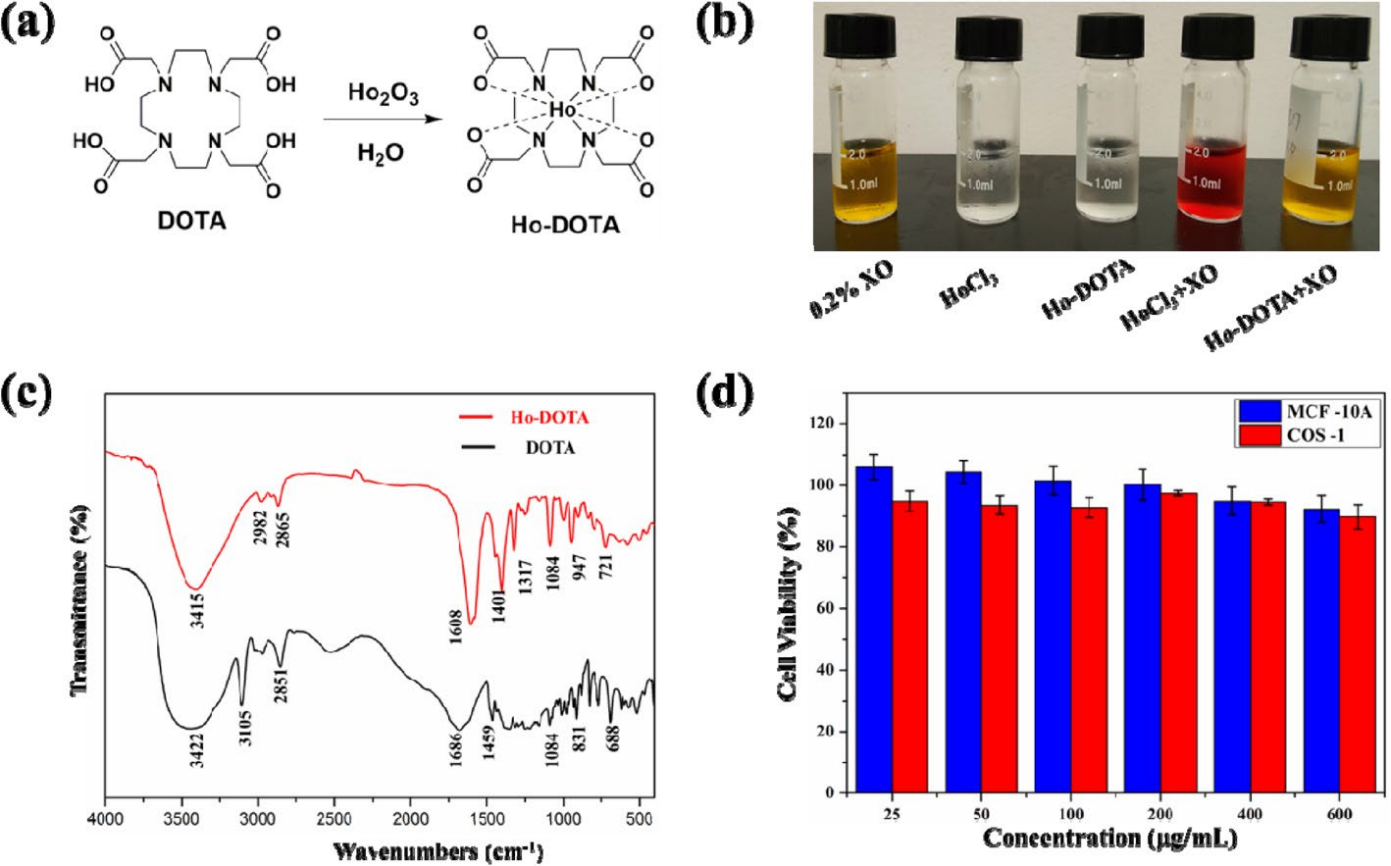
**a** Schematic representation of the synthesis of Ho-DOTA; **b** Chemical stability: detecting the leakage of Ho3 + in Ho-DOTA and HoCl3 in PBS by using the XO indicator. The solutions from left to right are pure XO solution (0.2%), pure 0.01 mM HoCl3 solution, pure 10 mM HO-DOTA solution, 0.01 mM HoCl3 solution containing the XO indicator, and 10 mM HO-DOTA solution containing the XO indicator; **c** FTIR spectra of DOTA and Ho-DOTA; **d** Cellular viabilities of MCF-10A cells and COS-1 cells after being treated with Ho-DOTA (25, 50, 100, 200, 400, 600 μg/mL) for 24 h

The Ho-DOTA was synthesized via a one-pot method by the reaction of Ho_2_O_3_ and DOTA in water, and the content of Ho element in complex was quantified by ICP-OES to be 28.87%, the yield was as high as 95%, as presented in Fig. 1a. Then N-Methyl-D-glutamine was added into the Ho-DOTA solution to tune the pH value to 7.4 for further biological application. The morphology and size of the Ho-DOTA chelate was determined by TEM. As shown in Fig. S2, the Ho-DOTA chelate was spherical-like nanoparticles and a well-distributed particle size with a mean diameter of 5 nm. To prove the formation of the Ho chelate, the molecular structure of Ho-DOTA was measured via MALDI-TOF–MS. MALDI-TOF–MS calcd for C_16_H_24_HoN_4_O_8_^+^ [M + H]^+^, 566.097; found 566.094 (Fig. S3). The chemical stability of Ho-DOTA was investigated using the XO indicator (0.2%). The free Ho^3+^ and yellow XO can form a red complex even when the concentration of Ho^3+^ was as low as 0.01 mM. However, almost none of red complex was found in the mixture of XO and high concentration of Ho-DOTA (10 mM), which indicated there was no obvious leakage of Ho^3+^ (Fig. 1b). In addition, the DOTA and Ho-DOTA were analysed by FTIR. As shown in Fig. 1c, for pure DOTA, the peak at 1686 cm^−1^ is ascribed to the stretching vibration of C = O (-COOH). While in Ho-DOTA, the stretching and deformation vibration band of 1686 cm^−1^ disappeared and a new vibration peak at 1608 cm^−1^ was observed, validating the coordination of COO- with Ho^3+^, further demonstrating the formation of Ho-DOTA chelate.

Furthermore, to investigate the long-term solution stability, Ho-DOTA was dissolved in different media, such as saline solution, PBS, FBS, DMEM and RPMI-1640, and there was no precipitate or aggregates observed in their aqueous solutions during 7 and 14 days of storage. The outstanding colloidal solubility and chemical stability of Ho-DOTA are favourable for its biocompatibility (Fig. S4).

### Cytotoxicity assessment of Ho-DOTA complex

The cytotoxicity of Ho-DOTA was estimated by the CCK8 test. The viability of MCF-10A and COS-1 cells was measured after 24 h incubation with increasing concentrations of Ho-DOTA (25, 50, 100, 200, 400, 600 μg/mL). The cell cytotoxicity was represented by the percentage of cell viability in contrast with control cells. After incubation with increasing concentration of Ho-DOTA for 24 h, the cell viability of both the two types of cells were not affected even at a relatively high concentration (600 μg/mL) (Fig. 1d). These results suggested that Ho-DOTA had the low cytotoxicity as a small molecular contrast agent.

### Toxicity assessment in vivo

**Fig. 2:**
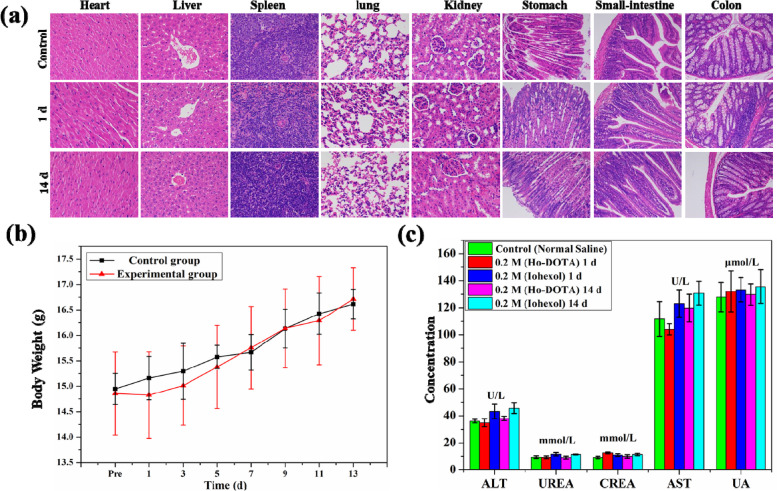
**a** H&E staining of main organs (heart, liver, spleen, lung, and kidney) and GI tract (stomach, small intestine, and colon) after intravenous administration of 200 μL NS or 0.2 M concentrations of Ho-DOTA after different time points (1 and 14 days); **b** Body weight fluctuations in mice injected with Ho-DOTA (*n* = 3) or treated with NS (*n* = 3) for 1d and 14 days; **c** Data was expressed as mean ± standard deviation. Typical biomarkers of liver and kidney function in mice injected with Ho-DOTA, iohexol and NS respectively (*n* = 3) for 1 day and 14 days. Data was expressed as mean ± standard deviation

The in vivo toxicity of Ho-DOTA was investigated by the histopathological analysis. After the injection of Ho-DOTA or iohexol via the tail vein on 1 day/14 days, no death occurred and no abnormal behaviours were found in the mice. For these mice, H&E staining showed the microstructures of important organs including cardiac muscle fibres, hepatic lobules, lymphoid follicles and germinal centre, alveoli, glomeruli and renal tubules, which were all regularly and neatly arranged at different time points (Fig. 2a and Fig. S5). For the mice carried out on Ho-DOTA orally at different time points, H&E analysis demonstrated that the mucosal, sub mucosal, and muscular structures of GI tract were clear and regulation without obvious histopathological damages (Fig. 2a).

Generally, no tissue necrosis, disorganization and inflammatory response were observed compared to the control group. In addition, the changes in body weight were measured every 2 days in different treatment groups (Fig. 2b). We found that body weight did not evidently decrease after the administration of Ho-DOTA, which further indicated its lower vivo toxicity as a small molecular contrast agent. Furthermore, before and after intravenous injection of Ho-DOTA or iohexol at different time points, representative biochemical indicators were employed to research the in vivo toxicity. The important indicators of liver function (AST, ALT) and vital biomarkers of kidney function (UREA, CREA and UA) at 1 and 14 days all proved no significant differences compared to the control group (Fig. 2c). However, some indicators in the group with iohexol were slightly higher than that of the control group and the group with Ho-DOTA, such as ALT, AST, and UA. These results further indicated that Ho-DOTA owned good biocompatibility and potential for in vivo application.

### Conventional CT and spectral CT imaging in vitro

**Fig. 3:**
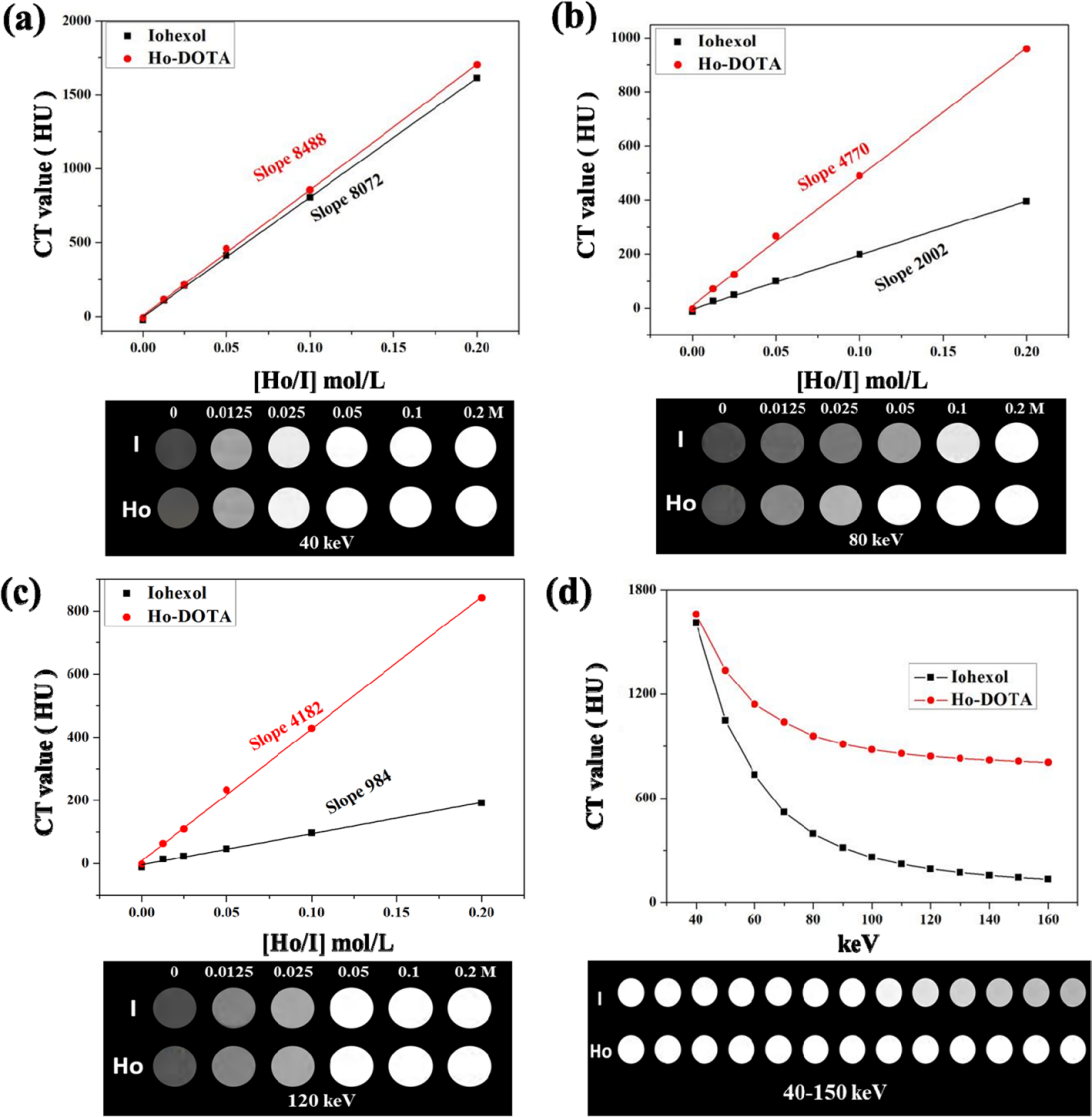
HU curves and spectral CT images of Ho-DOTA and iohexol at different concentrations (0, 0.0125, 0.025, 0.05, 0.1 and 0.2 M Ho or I) at **a** 40 keV; **b** 80 keV; **c** 120 keV. **d** Different mono-chromatic energies

The X-ray absorption capacity of Ho-DOTA and clinical iohexol was determined by spectral CT presenting in the black-and-white degree of CT images and Hounsfield unit (HU) values (Fig. 3). With the ascending equivalent element concentrations of Ho-DOTA and iohexol in monochromatic energies from 40 to 120 keV, the brightness of CT images for Ho-DOTA in each tube was higher than the corresponding images for the iohexol tube and the CT value are bigger than at the same concentration of the iohexol tube (Fig. 3a-c). As shown in Fig. 3c, at 120 keV, the value of the linear slope for Ho-DOTA was 4182, which was much higher than that of iohexol (984). Simultaneously, among the same monochromatic energy, the CT values linearly increased for both Ho-DOTA and iohexol in a concentration-dependent manner (Fig. 3a-c). Moreover, the HU curve trend of Ho-DOTA and iohexol at same concentration (0.2 M) represented their X-ray attenuation capacity with the ascending monochromatic energies from 40 to 160 keV (Fig. 3d). It further demonstrated the more powerful X-ray attenuation capability of Ho-DOTA directly. These results indicated the superiority of Ho-DOTA compared to clinical iohexol in spectral CT imaging.

### In vivo pharmacokinetics and biodistribution of Ho-DOTA complex

First, the BABL/C female mice were intravenously injected with 200 μL of 0.2 M Ho-DOTA and the same dosage of iohexol as a control. In the Ho-DOTA group and iohexol group, with 1 to 5 min, the renal collection system gradually emerged. The enhanced CT signal in the Ho-DOTA group decreased and gradually cleared away 1 h later (Fig. S6a). However, the enhanced CT signal in the control group declined after 5 min and gradually became no enhancement 30 min later (Fig. S5b). At the same time, the CT signal of the bladder was progressively augmented, proving that Ho-DOTA was metabolized by the urinary system and can be quickly cleaned up in vivo like clinical iohexol. For the mice which were administered 200 μL of iohexol via the caudal vein with equivalent concentration, the time for their collecting system of kidney and bladder lighted up was close to the Ho-DOTA group. Nevertheless, CT values in kidney after administration of Ho-DOTA at 1 and 5 min were higher than those the iohexol group, and the enhancement time were logger than the control group (Fig. S6a-b). From the above experiment, the CT enhancement effect of Ho-DOTA was better than that of iohexol at the same concentration.

To further investigate the superior X-ray attenuation capability, we executed CT imaging using Ho-DOTA and iohexol at a lower concentration (0.1 M) (Fig. S6c-d). We could see that the development of the enhanced CT imaging in the area of interest (kidney) was similar to those using Ho-DOTA and iohexol at a higher concentration (0.2 M). In comparison, a renal collecting system of the control group performed insufficiently and displayed a much weaker contrast enhancement for the inferior X-ray attenuation ability of clinical iohexol (Fig. S5d). Generally, at the lower concentration, Ho-DOTA had still more significance contrast enhancement than iohexol in our studies, which indicated that Ho-DOTA could be promisingly used in clinic as a high-performance and great-potential CT control agent.

### In vivo CT imaging of the GI tract

**Fig. 4:**
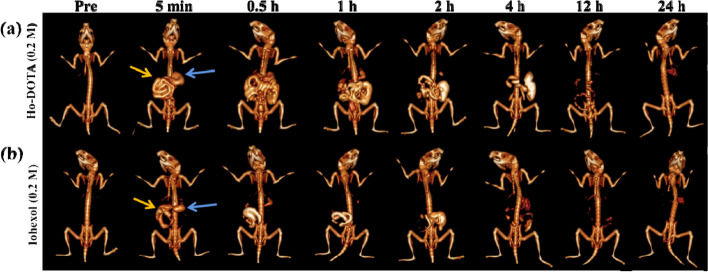
In vivo GI tract CT imaging after oral administration of (**a**) 0.2 M Ho-DOTA, (**b**) 0.2 M iohexol (blue arrows represent stomach and yellow arrows represent small intestine)

The CT imaging of the GI tract was performed by using Ho-DOTA (0.2 M) and iohexol (0.2 M) to investigate the outstanding spectral CT imaging ability (Fig. 4). At 5 min after oral 200 μL of Ho-DOTA (0.2 M) and iohexol (0.2 M), the stomach, duodenum, and proximal jejunum were gradually emerged with the filling of contrast agent. The signal of the stomach gradually became poorer and the outlines and shapes of the small intestine loop were more delineated 30 min later. After oral administration 1 h, the performance of the whole jejunum and ileum were bright while the stomach was further degraded. The morphology and sequence of GI tract were clearly outlined by Ho-DOTA, which would provide new thinking for the diagnosis of the gastrointestinal disease in contrast agents. After 12 h, we observed that most complex was emptied from the upper GI tract, and then absolutely excreted from the vivo after 24 h (Fig. 4a). In the control group at the same administration, the performacne of GI tract imaging was lack of continuity and integrality (Fig. 4b). Not surprisingly, the perfusion effect of iohexol had disadvantage in imaging upper GI compared to Ho-DOTA, which caused by its lower X-ray absorbance ability.

### In vivo spectral CT imaging of the GI tract

**Fig. 5:**
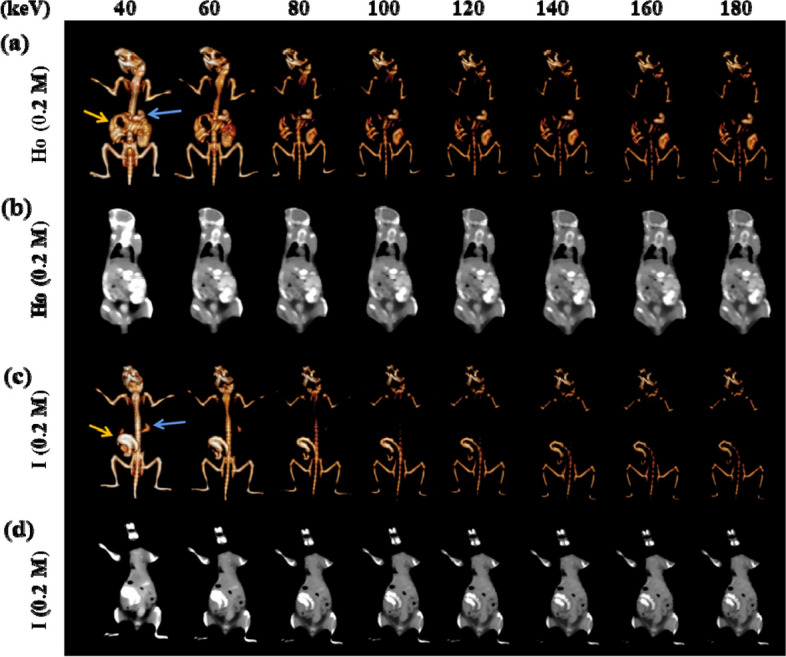
In vivo spectral GI tract CT imaging under different energies after using oral administration 0.2 M Ho-DOTA and iohexol at 0.5 h in healthy mice respectively (Blue arrows represent stomach and yellow arrows represent small intestine). **a** 3D reconstruction images using Ho-DOTA at different monochromatic energies (**b**) Coronal CT imaging using Ho-DOTA at different monochromatic energies. **c** 3D reconstruction images using iohexol at different monochromatic energies. **d** Coronal CT imaging using iohexol at different monochromatic energies

For spectral CT imaging, the healthy mice were scanned at 0.5 h after oral administration of 200 μL Ho-DOTA and iohexol (0.2 M Ho and I), respectively (Fig. 5). Different monochromatic energies (40, 60, 80, 100, 120, 140, 160 and 180 keV) of common and 3D reconstruction images were acquired by using the workstation. At all energies, the imaging brightness of Ho-DOTA in the GI tract was superior to that of iohexol. The outline of the stomach and other tissues can perform relatively high CT signals and be more clearly showed in the group with Ho-DOTA at lower energy (40–60 keV). With the increasing of the monochromatic energies (80–180 keV), iohexol and the tissues both declined sharply in the X-ray attenuation (140–180 keV). However, the contrast effect was excellent in imaging with high signal-to-noise due to the slightly signal decay of Ho-DOTA. Totally, compared to the clinical iodinate agent, Ho-DOTA was successfully imaged in the GI tract in our experiments, which manifested that it could serve for all kinds of biomedical applications as a high-performance CT contrast agent.

### In vivo CT imaging in mouse model of colitis

**Fig. 6:**
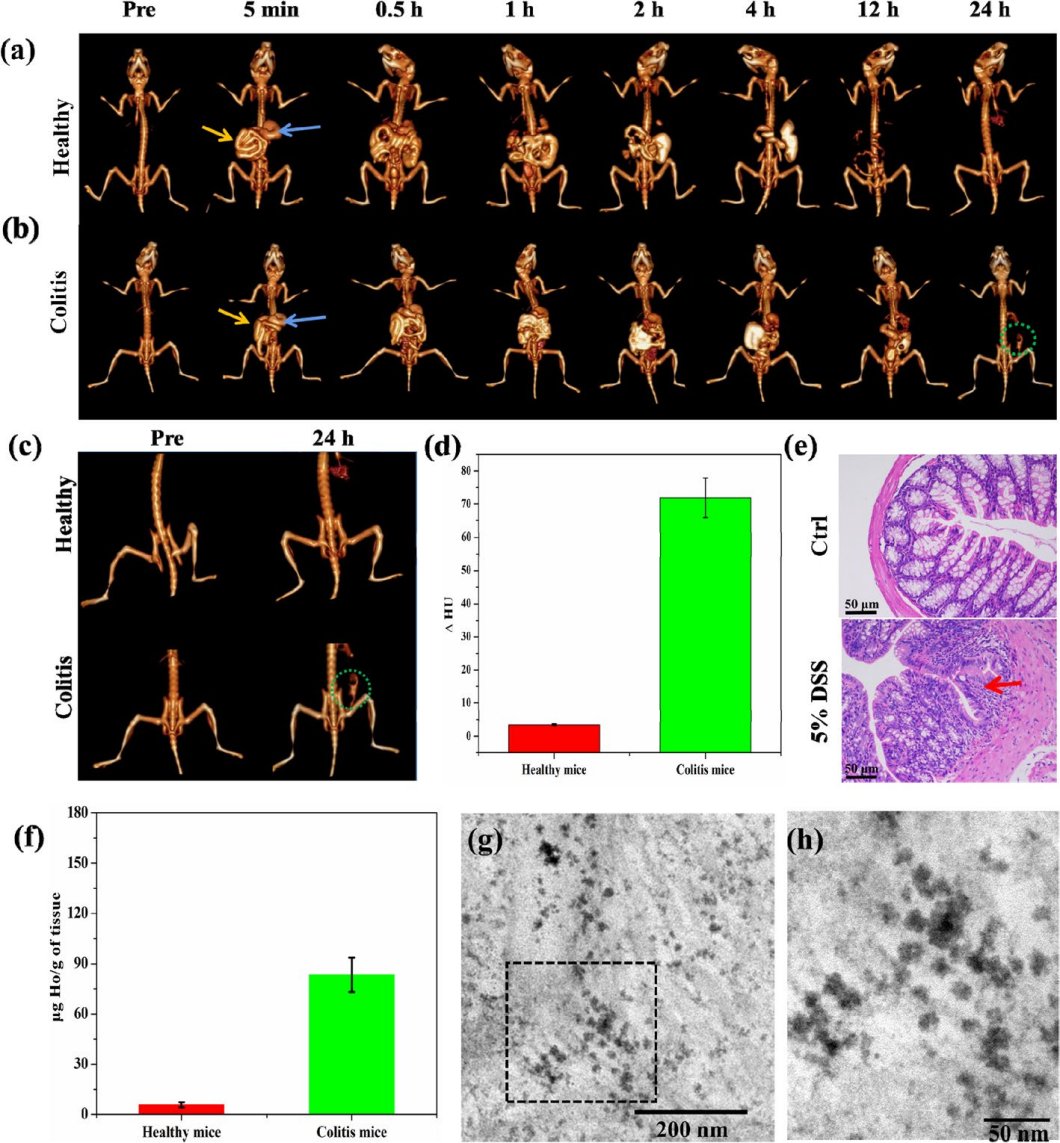
In vivo CT imaging of DSS-induced colitis mice and healthy mice administered Ho-DOTA, and Ho-DOTA accumulated in the inflamed large intestines of DSS-colitis mice. **a** Representative CT images of healthy mice, pre- and post-oral administration of Ho-DOTA. **b** Representative CT images of colitis mice, pre- and post-oral administration of Ho-DOTA (Blue arrows represent stomach and yellow arrows represent small intestine, green circle indicates Ho-DOTA accumulation in an area of colitis). **c** Representative CT images of healthy or colitis mice at pre and 24 h post oral administration of Ho-DOTA (Yellow circle indicates Ho-DOTA accumulation in an area of colitis). **d** Graph showing CT attenuation in the large intestines of healthy and DSS-colitis mice at the 24 h time point. **e** The H&E staining of control group’s colon section and 5% DSS group’s colon section (scale bar: 50 µm) (Red arrow: lamina intestinal gland disappeared and repalced by hyperplasticn connective tissue). **f** Holmium contents in the large intestines of healthy and colitis mice at 24 h post administration of Ho-DOTA by ICP-OES test. **g** Representative electron micrographs depicting the accumulation of Ho-DOTA in the large intestine of a DSS-colitis mouse at 24 h. **h** Magnification of the area inside the black square in panel (g). experiment, the CT enhancement effect of Ho-DOTA was better than that of iohexol at the same concentration

**Fig. 7:**
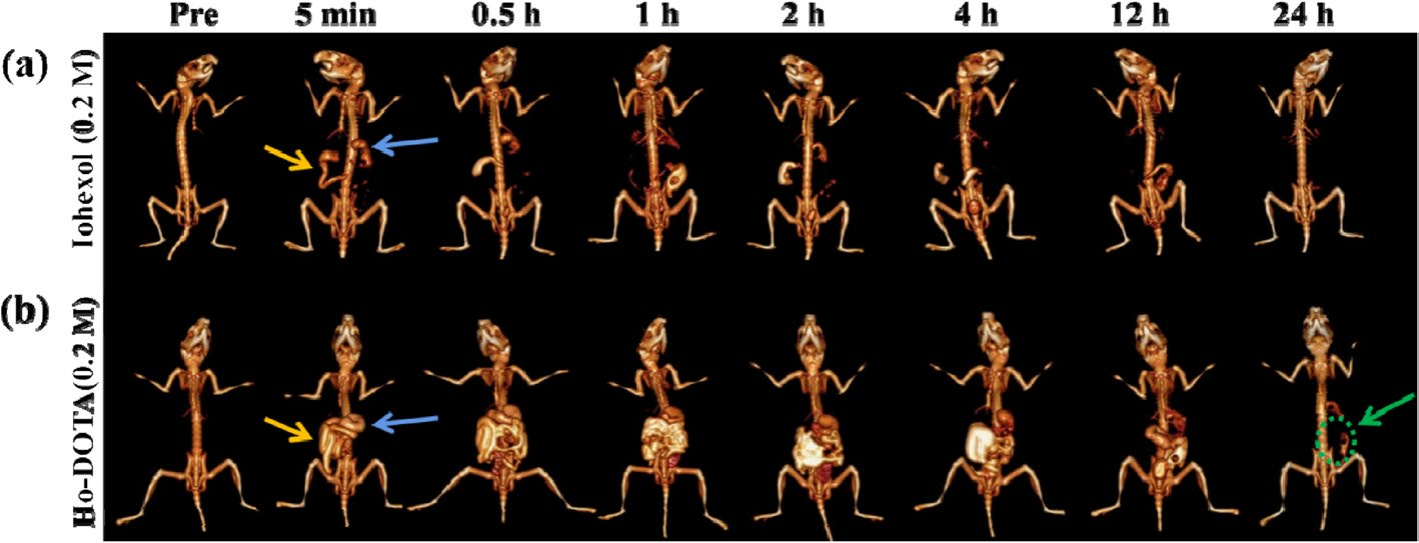
In vivo CT imaging of DSS-induced colitis mice administered Ho-DOTA or iohexol. Representative CT images of colitis mice, pre- and post-oral administration of (**a**) Ho-DOTA and (**b**) iohexol (Blue arrows represent stomach and yellow arrows represent small intestine, green arrow and circle indicates Ho-DOTA or iohexol accumulation in an area of colitis)

To explore the feasibility of imaging IBD using Ho-DOTA, the colitis mice were scanned at different time points after oral administration of 200 μL Ho-DOTA and iohexol (0.2 M Ho and I) owing to the promising prospect in vitro and vivo results. As shown in Fig. 6a, the enrichment rate of Ho-DOTA in colitis mice was similar to the healthy mice. We can see the complete gastrointestinal tract profile in healthy and colitis mice. Nevertheless, no CT signal was shown in the small intestines and large intestines of the healthy mice after 24 h post administration, while obvious accumulation was observed in colitis mice (Fig. 6a-b). Simultaneously, we could see that the CT signal attenuation in the area of the large intestines in the DSS-colitis mice were higher than healthy mice at the 24 h time point (Fig. 6c-d). To confirm the degree of inflammation of the DSS-colitis mice, the pathological sections of colon tissues were carried out. As demonstrated in Fig. 6e, the structure disruption of the native gland and infiltration of the inflammation cells was severe in colitis group and negligible in control group. In addition, we found that a significant amount of Holmium in the large intestine of colitis mice via ICP-OES test, which was higher than that in the healthy mice group (Fig. 6f). These ICP-OES results supported our observations from in vivo CT imaging that Ho-DOTA accumulated in the large intestine of colitis mice at 24 h post administration (Fig. 7b). Furthermore, we also investigated the accumulation of the chelate in the large intestine of DSS-colitis mouse via TEM, observing that the Ho-DOTA chelate accumulated in the large intestine of DSS-colitis mice (Fig. 6g-h). These results suggested that Ho-DOTA can serve as an excellent CT imaging agent for visualizing the GI tract and IBD due to its higher X-ray absorbance capability and specific distribution in vivo.

**Fig. 8:**
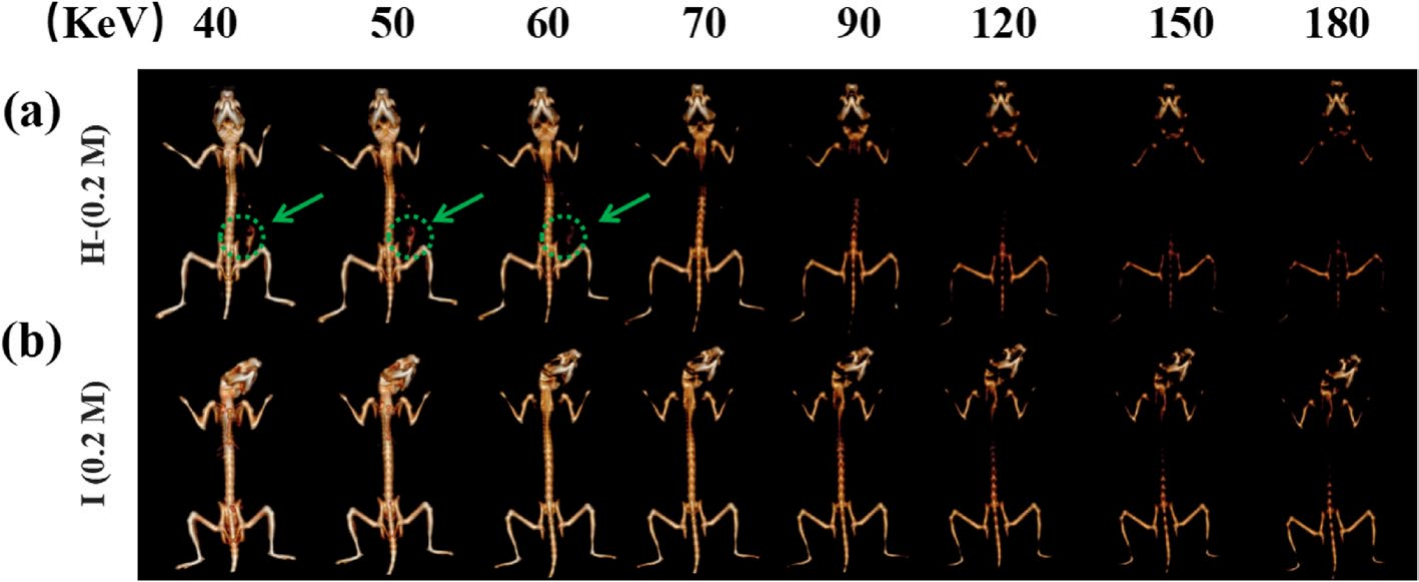
In vivo spectral GI tract CT imaging under different energies after using 0.2 M Ho-DOTA or iohexol at 24 h in colitis mice. **a** 3D reconstruction images using Ho-DOTA at different monochromatic energies in colitis mice. **b** 3D reconstruction images using iohexol at different monochromatic energies in colitis mice (Green arrow and circle indicates Ho-DOTA or iohexol accumulation in an area of colitis)

To further explore the efficiency of iohexol and Ho-DOTA in imaging inflammatory bowel diseases, in vivo CT imaging with Ho-DOTA and iohexol was performed with DSS-induced colitis mice (Fig. 7). The contrast agent display range of the gastrointestinal tract in the colitis mice using Ho-DOTA was broader than that in the control group administrated by iohexol. Compared with the colitis mice using iohexol, the contrast agent display range of the inflammatory intestinal tract in the colitis mice using Ho-DOTA was clearly observed at the 24 h time point. At the same time points, little CT contrast was performed in the corresponding posion of the colitis mice using iohexol. This suggested that Ho-DOTA has superior CT imaging ability than iohexol. For spectral CT imaging, the mice were scanned at 24 h after oral administration of 200 μL Ho-DOTA and iohexol in colitis mice. Different monochromatic energies (40, 50, 60, 70, 90, 120, 150 and 180 keV) of 3D reconstruction images were acquired by using the workstation (Fig. 8). The CT signals of Ho-DOTA accumulation in an area of colitis can be clearly observed at lower monochromatic energy (40–60 keV). With the increasing of the monochromatic energy (70–180 keV), the X-ray attenuation in the other tissues declined sharply. These results demonstrated that Ho-DOTA has higher sensitivity in the diagnosis of digestive system diseases by spectral CT imaging than convention CT imaging.

## Discussion

Gastrointestinal contrast agents that can be used in clinical practice should have the advantages of easy synthesis, low cost, high stability, biological safety, and strong X-ray attenuation ability. Although Bi-DTPA has been reported as a high-performance CT contrast agent [33], but the linear chelating ligand DTPA may increase the content of free metal ions and thus lead to easy deposition in the kidney and bone due to the unstable linear structure [54]. However, macrocyclic chelating ligand DOTA, as a closed ligand, can bind tightly to the metal ion, and form more stable complexes [52]. In addition, although Dex-Ce NPs have been reported as a potential CT contrast agent for imaging GIT with IBD [37], the low K-edge of Cerium (40.4 keV) leads to a very weak energy-dependent attenuation character of Dex-Ce NPs, which achieves poor performance for spectral CT imaging. Therefore, it’s urgently needed to develop contrast agents with a stable structure and suitable imaging element for gastrointestinal spectral CT imaging of IBD. On the basis of our previous study [35], we synthesized the small-molecule contrast agent Ho-DOTA (Ho’s k-edge is 56 keV) and studied its multiple properties through a series of methods.

First, we prepared Ho-DOTA in one-pot method under mild conditions and confirmed the structure of Ho-DOTA by TEM, MALDI-TOF–MS, ICP-OES, and FT-IR analyses. Moreover, the chemical stability of Ho-DOTA was determined by studying long-term storage of Ho-DOTA solutions with different media and using the XO indicator (0.2%). We found that Ho-DOTA had the advantages of easy synthesis, high yield (95%), low cost, and outstanding solubility and chemical stability, which laid a foundation for industrial production.

Then, in vitro CCK-8 studies, 600 μg/mL of Ho-DOTA had little effect on the proliferation of normal MCF-10A and COS-1 cells. Like all the other heavy metals, high dose/long-term exposure may lead to adverse effects, but the rapid renal and gastrointestinal clearance of Ho-DOTA ensures its low biological toxicity. In addition, a series of in vivo toxicity assessment experiments, including pharmacokinetics and biodistribution of Ho-DOTA were further performed to demonstrate its good biocompatibility and its potential for in vivo application.

CT imaging is crucial for the diagnosis and treatment of IBD patients, while the emerging spectral CT imaging improves the imaging sensitivity of in vivo imaging based on the strong X-ray attenuation ability of high atomic number elements [25, 55]. In this study, the results of conventional CT and spectral CT imaging in vitro showed that the CT value of the same concentration of Ho-DOTA was higher compared to clinically approved iodinated CT contrast media, indicating that Ho-DOTA had excellent CT imaging ability as a high-performance contrast agent. For in vivo GI imaging, the GI lumen depicted by Ho-DOTA showed a significantly enhanced signal at any energy (40–180 kev). In addition, for the in vivo CT imaging of DSS-induced colitis mice and healthy mice, Ho-DOTA had more aggregation in the inflamed site of the colon than iohexol, and showed a more stable attenuation effect, which demonstrated the superior spectral CT imaging capability of Ho-DOTA in the inflammatory intestinal tract. Besides, we then investigated the accumulation of the Ho-DOTA chelate in the large intestine of DSS-colitis mouse via TEM and ICP-OES test, which supported our observations from in vivo CT imaging that Ho-DOTA accumulated in the large intestine of colitis mice at 24 h post administration. Moreover, the colon H&E staining images obtained in colitis mice and normal mice after imaging further confirmed the spectral CT imaging of Ho-DOTA provided much more diagnostic sensitivity and accuracy in the inflammatory intestine. In conclusion, our study clearly demonstrated the imaging potential of Ho-DOTA as an excellent gastrointestinal spectral CT contrast agent.

Of course, this study also has some limitations. First, a small animal colitis model was used in our study rather than large animal models, as large animal models may better mimic human disease. Furthermore, although adverse effects of these drugs are not observed, repeated and higher dose studies, and longer term studies are needed further to clearly determine in vivo safety. We will aim to further systematically evaluate the long-term toxicity and spectral CT imaging capabilities of Ho-DOTA in mammals and primates and advance the potential future clinical applications of Ho-DOTA.

## Conclusion

In summary, we reported a novel spectral CT contrast agent Ho-DOTA with a facile one-step synthesis method. Ho-DOTA showed great water solubility, excellent chemical stability, lower cytotoxicity, and outstanding X-ray attenuation for in vitro and vivo spectral CT imaging. In the above these studies, Ho-DOTA performed strong CT perfusion efficiency in the GI and accumulated in colon affected by inflammation. Notably, most oral doses were emptied from the in vivo within 24 h in healthy mice. It showed that Ho-DOTA could be removed with gastrointestinal peristalsis without causing adverse effects on organ function. Therefore, it is of great significance that Ho-DOTA serves as a potential high-performance CT contrast agent for GI imaging. Furthermore, due to the energy-dependent attenuation character of Ho-DOTA, a conspicuous GI imaging could be achieved with a low Ho-DOTA oral dose by using the appropriate monochromatic energy of spectral CT, which was conducive to increase diagnostic sensitivity and accuracy of IBD. Overall, our studies provide a promising prospect for the development of a novel small molecular lanthanide complex as a spectral CT contrast agent with clinical transformation potential.

### Supplementary Information

**Additional file 1: Fig. S1. **Body weight fluctuations in DSS mice (*n* = 3) or healthy mice (*n* = 3) for 7 days. Data was expressed as mean ± standard deviation.** Fig. S2. **(a, b) TEM images of the as-prepared Ho-DOTA. (c) The size distribution histograms of Ho-DOTA. The particle size distribution of Ho-DOTA, counted from 260 nanoparticles shown in typical TEM images, showing these nanoparticles are with small size and their particle sizes were relatively uniform. **Fig. S3. **MALDI-TOF-MS of Ho-DOTA. MALDI-TOF-MS calcd for C16H24HoN4O8+ [M+H]+, 566.097; found 566.094. **Fig. S4. **The stability of Ho-DOTA in different media (100 mg/mL, from left to right: NaCl, PBS, FBS, DMEM and RPMI-1640) at 37 °C for 7 (a) and 14 days (b). **Fig. S5. **Hematoxylin and eosin (H&E) staining of important organs for normal mice at different time points after the injection of iohexol (0.2 M) via the tail vein.** Fig. S6.** In vivo CT urography imaging using Ho-DOTA and iohexol (Blue arrows represent kidney and yellow arrows represent bladder). CT imaging after intravenous administration of (a) 0.2 mol/L Ho-DOTA, (b) 0.2 mol/L iohexol, (c) 0.1 mol/L Ho-DOTA and (d) 0.1 mol/L iohexol.

## Data Availability

Data will be made available on request.
