# Supplementary material for: Achieving safe and high-performance gastrointestinal tract spectral CT imaging with small-molecule lanthanide complex
Source: Biomater Res. 2023 Nov 22;27:119. doi: 10.1186/s40824-023-00463-x (PMC10664581; doi:10.1186/s40824-023-00463-x)
Supplement: Supplementary file 1 — Additional file 1: Fig. S1. Body weight fluctuations in DSS mice (n = 3) or healthy mice (n = 3) for 7 days. Data was expressed as mean ± standard deviation. Fig. S2. (a, b) TEM images of the as-prepared Ho-DOTA. (c) The size distribution histograms of Ho-DOTA. The particle size distribution of Ho-DOTA, counted from 260 nanoparticles shown in typical TEM images, showing these nanoparticles are with small size and their particle sizes were relatively uniform. Fig. S3. MALDI-TOF-MS of Ho-DOTA. MALDI-TOF-MS calcd for C16H24HoN4O8+ [M+H]+, 566.097; found 566.094. Fig. S4. The stability of Ho-DOTA in different media (100 mg/mL, from left to right: NaCl, PBS, FBS, DMEM and RPMI-1640) at 37 °C for 7 (a) and 14 days (b). Fig. S5. Hematoxylin and eosin (H&E) staining of important organs for normal mice at different time points after the injection of iohexol (0.2 M) via the tail vein. Fig. S6. In vivo CT urography imaging using Ho-DOTA and iohexol (Blue arrows represent kidney and yellow arrows represent bladder). CT imaging after intravenous administration of (a) 0.2 mol/L Ho-DOTA, (b) 0.2 mol/L iohexol, (c) 0.1 mol/L Ho-DOTA and (d) 0.1 mol/L iohexol. [file 40824_2023_463_MOESM1_ESM.docx]

Supplementary information for

**Achieving safe and high-performance gastrointestinal X-ray imaging with small-molecule lanthanide complex**

Xiaoling Che^†^, Chunmei Yang^†^, Liping Pan^†^, Didi Gu, Guidong Dai, Jian Shu* and Lu Yang*

*Department of Radiology, The Affiliated Hospital of Southwest Medical University, Luzhou, 646000, People’s Republic of China*

*^†^These three authors contributed equally to this work*

**Correspondence to: Jian Shu, shujiannc@163.com; Lu Yang, yanglu@swmu.edu.cn*

**Materials and instruments**

Starting materials were used without purification which was purchased from Bide Pharmatech Ltd. (Shanghai, China), Aladdin Reagent Co. Ltd (Shanghai, China), Beyotime Biotechnology (Shanghai, China) and GIBCO (Thermo Fisher Scientific, Waltham, MA, USA). The Fourier Transform infrared (FTIR) analysis of Ho-DOTA was performed on an IR Affinity-1S spectrometer (Shimadzu, Japan). Mass spectrum analysis of Ho-DOTA (without N-Methyl-D-glutamine) was performed using MALDI-TOF (Bruker, Ettlingen, Germany). The content and composition of Ho in the prepared Ho-DOTA was determined by ICP-OES instrument (Agilent, Santa Clara, USA).

**Supplementary Figures**


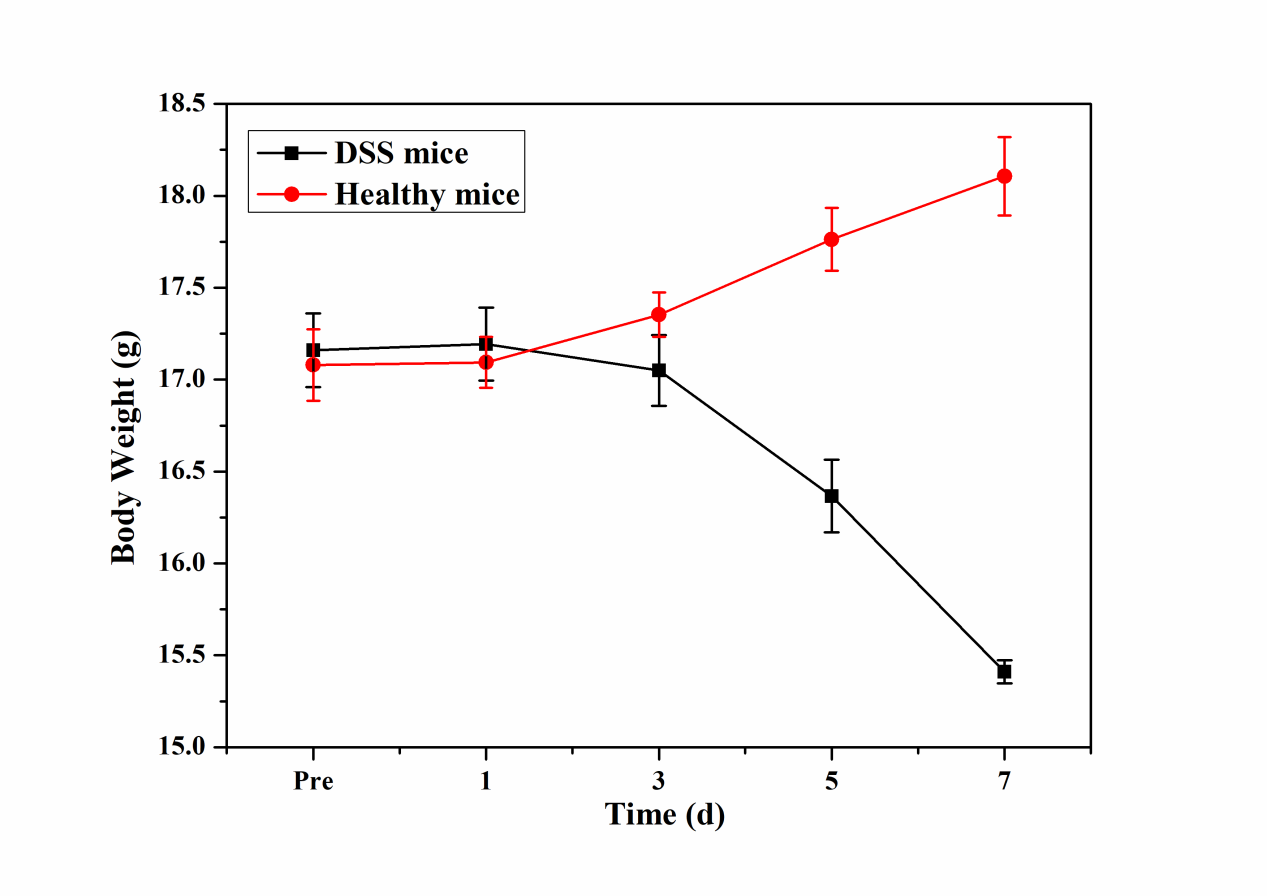


**Fig. S1** Body weight fluctuations in DSS mice (n = 3) or healthy mice (n = 3) for 7 days. Data was expressed as mean ± standard deviation.


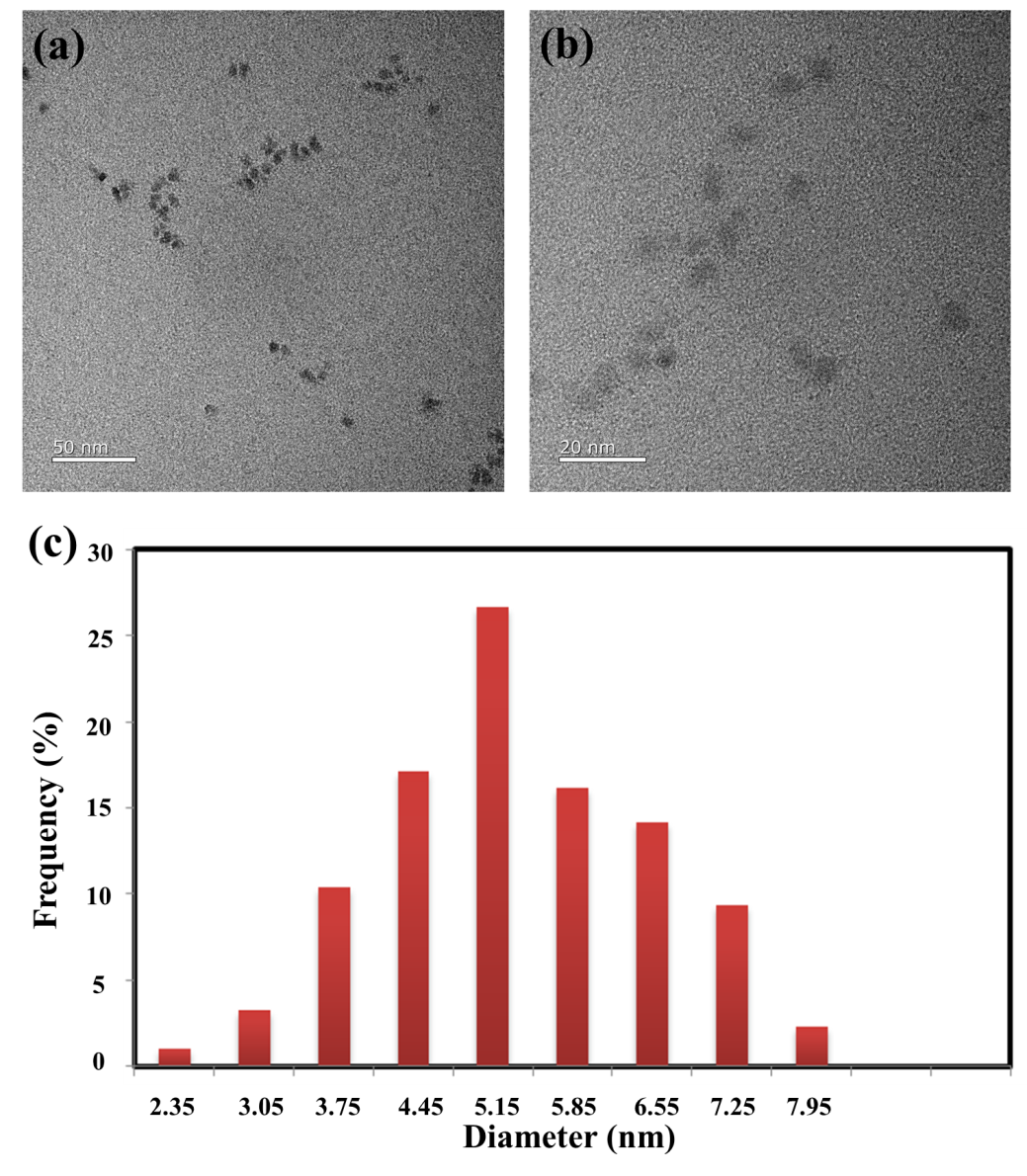


**Fig. S2** (a, b) TEM images of the as-prepared Ho-DOTA. (c) The size distribution histograms of Ho-DOTA. The particle size distribution of Ho-DOTA, counted from 260 nanoparticles shown in typical TEM images, showing these nanoparticles are with small size and their particle sizes were relatively uniform.


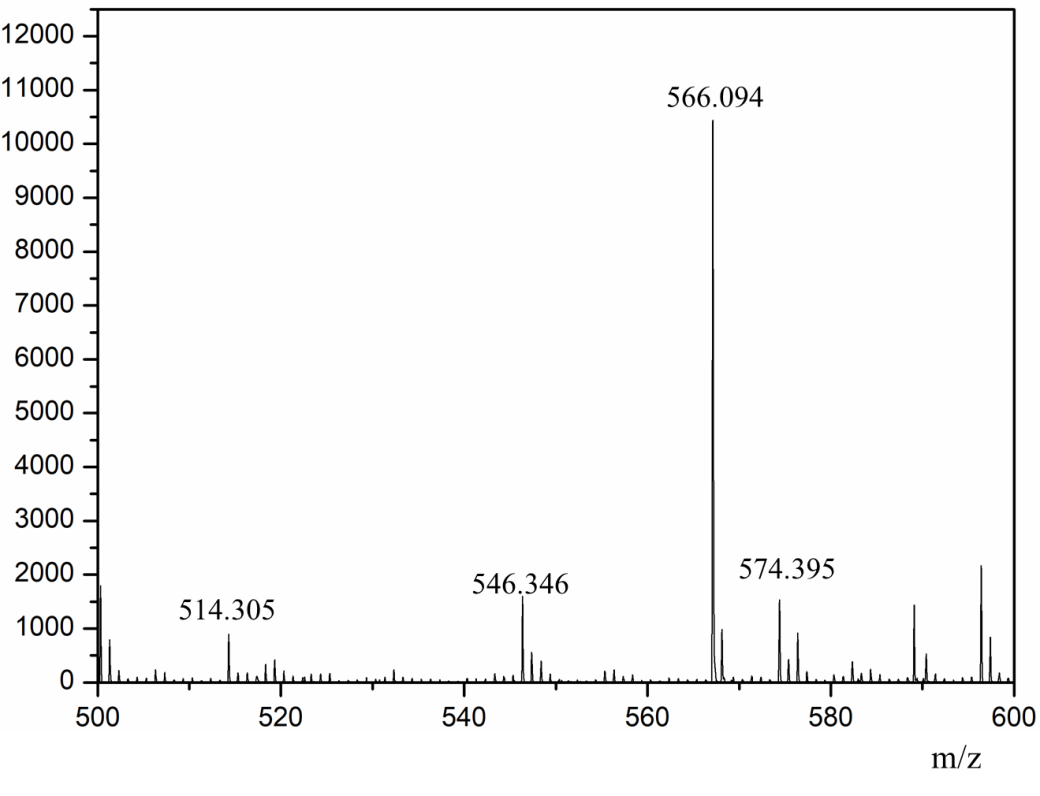


**Fig. S3** MALDI-TOF-MS of Ho-DOTA. MALDI-TOF-MS calcd for C_16_H_24_HoN_4_O_8_^+^ [M+H]^+^, 566.097; found 566.094.


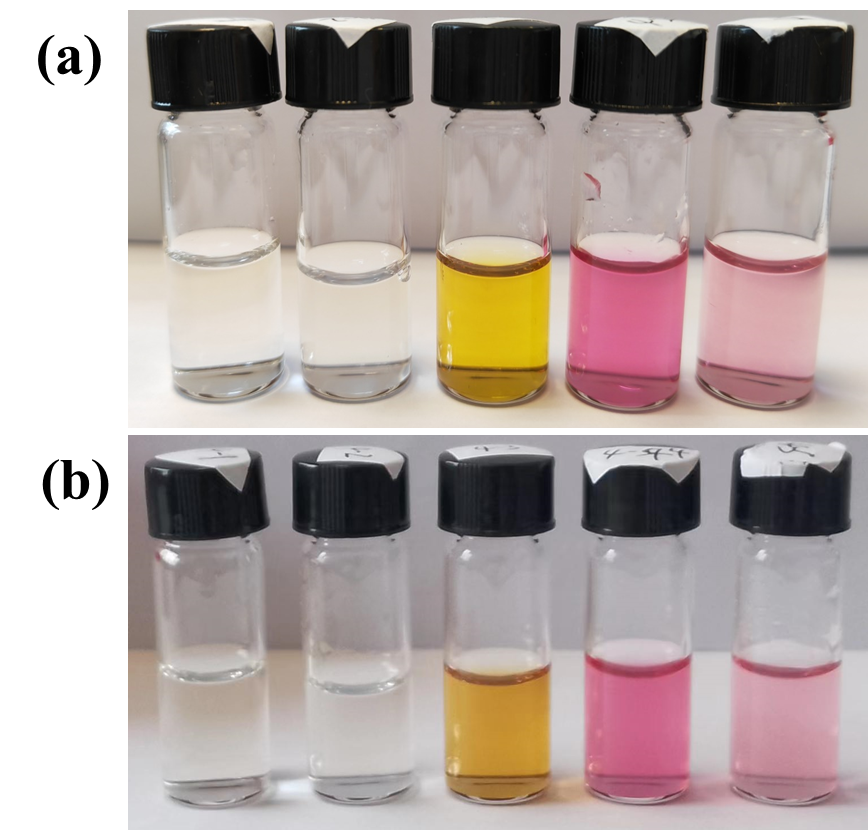


**Fig. S4** The stability of Ho-DOTA in different media (100 mg/mL, from left to right: NaCl, PBS, FBS, DMEM and RPMI-1640) at 37 °C for 7 (a) and 14 days (b).


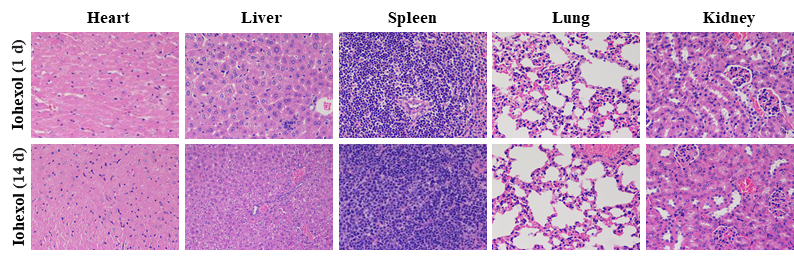


**Fig. S5** Hematoxylin and eosin (H&E) staining of important organs for normal mice at different time points after the injection of iohexol (0.2 M) via the tail vein.


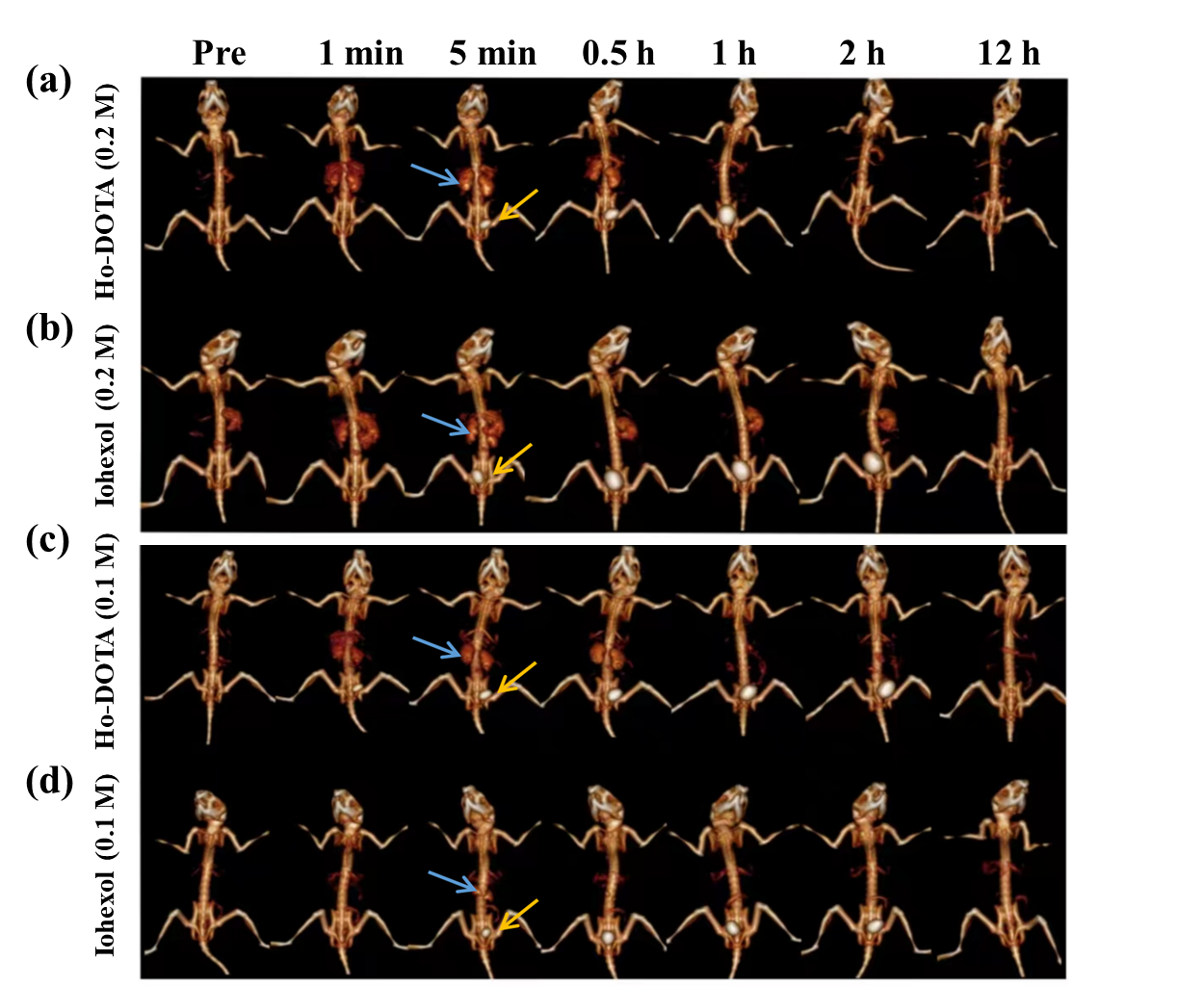


**Fig. S6** In vivo CT urography imaging using Ho-DOTA and iohexol (Blue arrows represent kidney and yellow arrows represent bladder). CT imaging after intravenous administration of (a) 0.2 mol/L Ho-DOTA, (b) 0.2 mol/L iohexol, (c) 0.1 mol/L Ho-DOTA and (d) 0.1 mol/L iohexol.
